# Quality of Life in Yoga Practitioners—Research Conducted on a Population of Polish Yogis

**DOI:** 10.3390/ijerph191912023

**Published:** 2022-09-23

**Authors:** Zuzanna Piekorz, Agnieszka Radzimińska, Andrzej Lewandowski, Roman Ossowski

**Affiliations:** 1 Department of Physiotherapy, Faculty of Health Sciences, Ludwik Rydygier Collegium Medicum in Bydgoszcz, Nicolaus Copernicus University in Toruń, 87-100 Toruń, Poland; 2 Institute of Psychology, Kazimierz Wielki University in Bydgoszcz, 85-064 Bydgoszcz, Poland

**Keywords:** CAM, complementary and alternative medicine, physical activity, hatha yoga

## Abstract

*Background:* The aim of the study is to determine the quality of life of a population of healthy adult yoga practitioners and the correlation between the features of yoga practice and the quality of life. *Methods:* A total of 300 people aged 35–50 years were examined and divided into two groups. The respondent (SG; *n* = 150) was a yoga practitioner, and the control (CG; *n* = 150) did not practice yoga. The original questionnaire and the quality-of-life questionnaire (WHOQOL-BREF) were used. The chi-square test, the Kolmogorov–Smirnov test, the Mann–Whitney U test, the ANOVA Kruskal–Wallis test and the linear regression model were used. The significance level was set at *p* < 0.05. *Results:* Yoga practitioners are characterised by a significantly greater overall satisfaction with the quality of life (U = 9794.50; *p* < 0.05), a higher level of health satisfaction (U = 9194.50; *p* < 0.01) and higher satisfaction with quality life in terms of the results of somatic domains (U = 7579.00; *p* < 0.001), psychological (U = 8554.00; *p* < 0.001) and environmental domains (U = 7919.5; *p* < 0.001). A relationship was observed between the experience of yoga practice and the assessment of the quality of life in terms of physical functioning. *Conclusions:* The practice of adult yoga positively influences the assessment of their quality of life in the physical, mental and environmental spheres.

## 1. Introduction

Initially, in pre-historic India, yoga served as a method for disease prevention and treatment [1]. Currently, natural methods of improving health, collectively referred to as complementary methods, are increasingly embraced by medicine [2]. Furthermore, scientists are keener on examining their use in the prevention and treatment of numerous diseases [3,4]. That trend is most prevalent in countries where the method originates from and in highly developed countries [4]. Unfortunately, in Central Europe, the therapeutic use of complementary methods and scientific verification of their effectiveness is negligible.

Even though yoga practice is growing in popularity in Central Europe and Poland, there is no reliable data on the number of practitioners [5,6]. “Polish yoga” is a physical activity practised to maintain well-being and it is considered a method of personal development rather than a form of relaxation therapy or a path of spiritual development [7]. In Poland, the most popular form of yoga is hatha yoga, which comprises physical exercises (*asanas*), breathing exercises (*pranayamas*), and relaxation techniques [1]. Despite the growing popularity of yoga in Poland, the number of polish studies on this topic is contrastingly low compared to the foreign scientific reports.

The literature on yoga shows that numerous practitioners across the world seek to apply yoga techniques to groups of healthy people, patients, and people in certain age groups [4]. Quality of life is a frequently assessed parameter, whose level seems to be positively associated with yoga practice [8,9,10,11]. 

Given the substantial number of studies on the quality of life in patients and the elderly who practise yoga, a small percentage of such studies conducted in healthy adults, and scarce research conducted in Central European countries, we decided to carry out the following study. The objective of this study is to estimate the quality-of-life level in adults practising yoga and to evaluate the correlations between the characteristics of yoga practice and the quality of life. These studies seem to be particularly useful, taking into account the fact that the yoga system in Poland is still treated with a high degree of criticism. It seems that the observations of the possible impact of yoga exercises on the body may significantly differ from the results of studies conducted in India or the USA.

## 2. Materials and Methods

### 2.1. Study Design

The study presents some results obtained in 2017 research conducted on a group of yoga practitioners in Poland. The Bioethics Committee of the Collegium Medicum in Bydgoszcz, Nicolaus Copernicus University in Torun (82/2017) has approved the study protocol. A total of 345 adults were screened for the study, but ultimately 45 people were excluded from the study due to one or more medical conditions or failure to meet the age criterion. The remaining 300 participants were divided into two equal groups—a study group (*n* = 150) comprised of yogis and a control group (*n* = 150) comprised of adults who did not practise yoga.

The study group: inclusion criteria were—voluntary consent to the study, ages of 35–50, general good health, and participant’s statement that they have been practising yoga for 2 h per week for at least a year. Study participants were recruited at yoga studios and facilities with *Iyengar yoga* [1] or academic yoga classes [12].

Control group: Inclusion criteria were—voluntary consent to the study, ages of 35–50, general good health, and participant’s statement that they have never practised yoga. Study participants were recruited at schools, educational institutions, offices, and medical facilities. The control group participants were recruited to reflect the socio-demographic characteristics of the study group.

### 2.2. Study Procedure

The study was conducted on an individual and group level. Study participants received surveys directly from the study investigator, yoga teacher/instructor, or picked them up at their yoga studio or workplace. The subjects received the following copies: information note on the study objective, study inclusion/exclusion criteria, instructions for filling out questionnaires, and consent to the study. Once the participants filled out the questionnaires manually, they returned them directly to the study investigator or their yoga teacher/instructor or submitted them at designated places.

### 2.3. Instruments

Interview questionnaire: Both the study group and the control group filled out Part 1 of the questionnaire which consists of 5 sociodemographic questions. The study group also filled out Part 2 of the questionnaire which consists of 6 questions on yoga practice. The quality-of-life questionnaire (WHOQOL-BREF) consists of 26 questions that assess quality of life in somatic, psychological, social, and environmental domains. Additionally, there are two questions on the assessment of the overall quality of life and general health [13,14].

### 2.4. Data Analysis

We analysed the collected data using several measures. The Chi-squared test was used to verify relationships between two qualitative variables, whereas the Kolmogorov–Smirnov test was used to examine the variable distribution. To compare rank distributions, we used the Mann–Whitney U-test in case of dichotomous grouping variables, or the ANOVA Kruskal–Wallis test in case of polytomous grouping variables. Furthermore, we complemented the ANOVA Kruskal–Wallis test with the Bonferroni correction to determine significant differences between the SG and the CG. For each analysis, the level of statistical significance was set to *p* < 0.05.

## 3. Results

Characteristics of the study participants are presented in Table 1 and Table 2.

The SG and the CG were significantly similar in terms of socio-demographic characteristics. The only difference was the participants’ education, and yogis with higher education represented a significantly larger group. 

**Figure 1:**
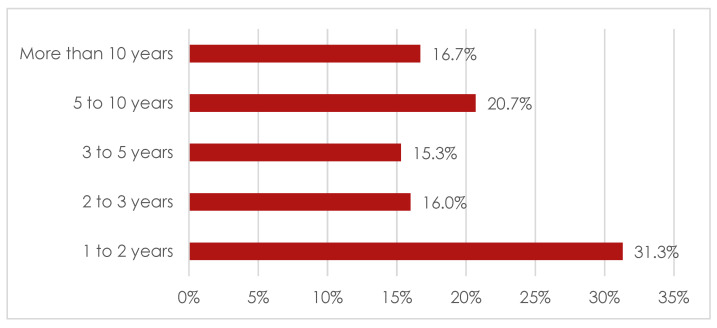
Graphic representation of yoga practice experience among practitioners.

**Figure 2:**
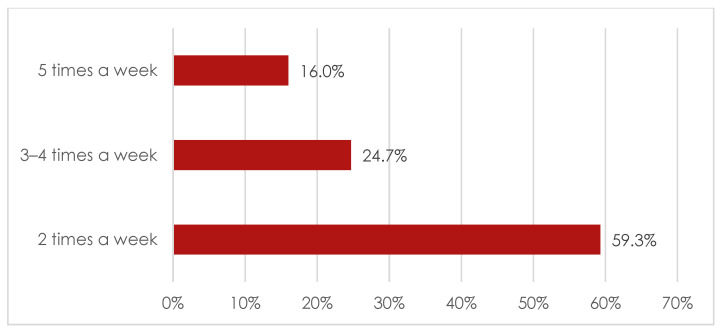
Graphic representation of frequency of yoga practice among practitioners.

Figure 1 and Figure 2 present characteristics of yoga practice.

As can be seen in the Figure 1 and Figure 2, over 31% of study participants reported that they have been practising yoga for 1–2 years, whereas over 20% of study participants have been practising yoga for 5–10 years. Majority of respondents confirmed that they practise yoga twice a week. 

Table 3 shows comparative characteristics in the following domains—quality of life, quality of life satisfaction, and own health satisfaction of both the SG and the CG. 

The table shows statistically significant differences between the study group and the control group in terms of evaluation of the quality of life in somatic, psychological, and environmental domains. Statistically significant differences were also observed in terms of quality-of-life satisfaction and own health satisfaction. Study group participants scored higher in all domains. However, there are no significant differences between the quality-of-life scores in the social domain.

**Figure 3:**
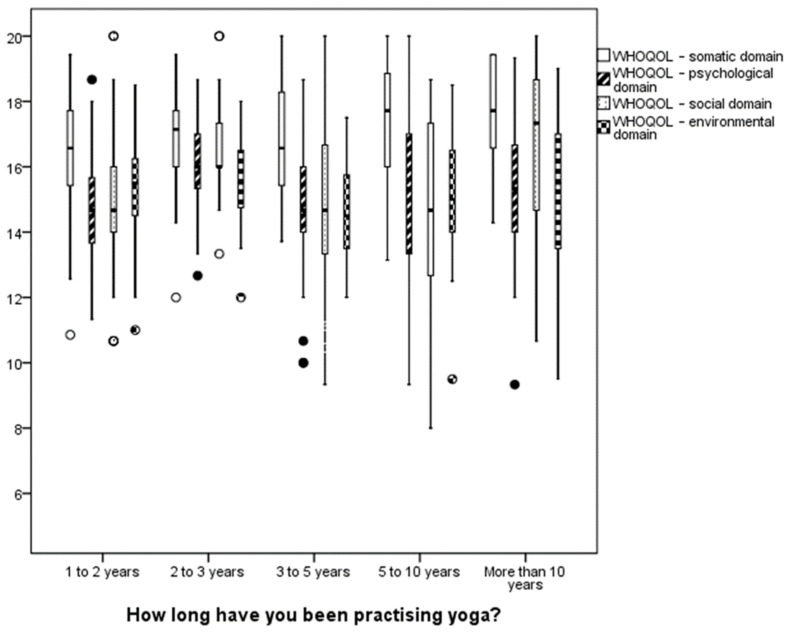
Graphic representation of quality-of-life domains of groups with varying yoga practice experience. Annotation. Lower boundary of the box—lower quartile; upper boundary of the box—upper quartile; line inside the box—median; o—observations from 1.5 to 3 quarter spacing from the box; •—outlier.

By analysing relations between the quality-of-life level and yoga practice characteristics, such as practice experience and frequency, the authors determined relationships between specific characteristics of the study group. The results are presented in Table 4 and Figure 3.

Statistically significant differences in terms of quality of life in the somatic, psychological, and social domains were observed in groups with varying yoga practice experience. This is further illustrated by Figure 3. 

We observed one statistically significant difference between groups that have been practising yoga for less than two years and for over ten years, and that is the quality-of-life score in the somatic domain (*p* = 0.031). 

Table 5 shows the impact of practice frequency on the quality-of-life assessment.

As the table shows, there are no statistically significant results for any of the variables. This means that the frequency of yoga practice did not impact the quality-of-life score among study participants.

**Figure 4:**
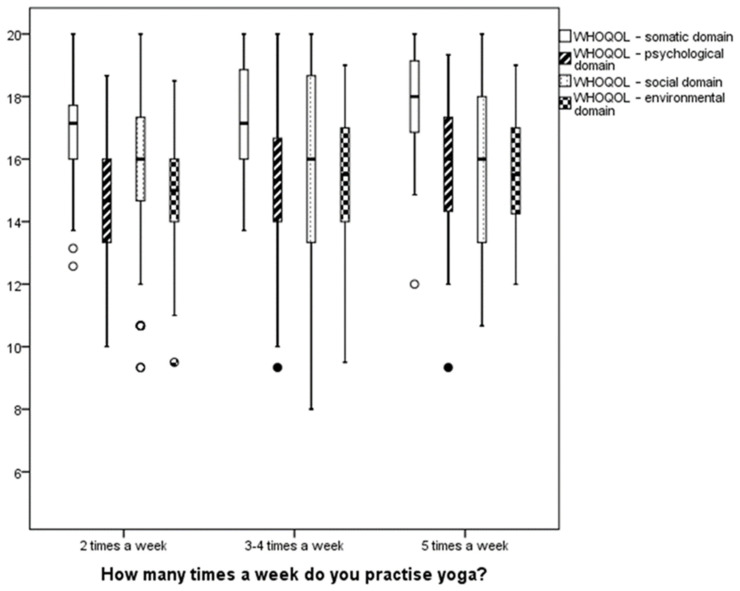
Graphic representation of quality-of-life domains of groups with varying frequency of yoga practice. Annotation. Lower boundary of the box—lower quartile; upper boundary of the box—upper quartile; line inside the box—median; o—observations from 1.5 to 3 quarter spacing from the box; •—outlier.

Figure 4 shows characteristics of quality-of-life domains in groups with varying frequency of yoga practice.

Scores in quality-of-life domains were similar across groups with varying frequency of yoga practice.

## 4. Discussion

The objective of this study was to assess quality of life in adults practising yoga. We tested our main hypothesis whether practitioners score higher in all quality-of-life domains. The obtained results positively verified the quality-of-life satisfaction, own health satisfaction, as well as quality of life in the somatic, psychological, social, and environmental domains. 

This result was not surprising given that the participants in the study group repeatedly reported that they consider their high quality of life to be closely related to their yoga practice. However, it should be remembered that the level education was the factor which also differed the study groups. It is possible that it is related to the level of quality of life of the respondents.

The available literature presents results of numerous studies on quality of life in yogis, majority of whom believe that there is a relationship between practising yoga and the quality-of-life level. The obtained results are consistent with the results of research conducted around the world, which indicate that the influence of yoga is universal and independent of culture, religion, and its social perception.

This research yielded significant differences in quality-of-life scores in the somatic domain between the yogis and the control group. Research on overweight and obese individuals [15], residents of nursing homes [16], and individuals diagnosed with diabetes [17] yielded similar results. Moreover, yogis also scored noticeably higher results in studies which used research tools other than WHOQOL-BREF to assess the quality of life [18,19,20,21,22,23,24,25]. The results presented above are mostly consistent with the results of our own research, which can be explained by the eightfold path of yoga. Each element constitutes a component in the complex learning of yoga, outlines the skills that one can acquire and provides methods to obtain them [1]. Study group participants practised *hatha yoga*, thereby also practising *asanas* and *pranayamas,* which are physical components of yoga practice. It would seem that there is a relationship between this activity for perfecting one’s body and a higher quality-of-life score in the somatic domain. This research revealed significant differences between the SG and the CG in the quality-of-life scores in the psychological domain. Research on yogis with diagnosed overweight and obesity [15], schizophrenia [26], diabetes [17], as well as yogis who are also residents of nursing homes [16] yielded similar results. Studies that used instruments other than the WHOQOL-BREF questionnaire showed a tendency toward higher quality-of-life scores in the psychological and mental health domains among yoga practitioners [20,22,23,27]. Thus, the results obtained in this research and in the available literature seem to confirm the multifaceted impact of yoga exercises. Even though only a few study participants used techniques from the mental dimension of yoga (*pratyahara*, *dharana*) as the leading form of practice, the relationship between yoga exercises and scores in the psychological domain is clearly noticeable. Finally, our analysis revealed a significant difference in the environmental domain, where yogis scored higher than the control group, which is consistent with data in the literature [15,16,20,27]. However, this tendency was not as pronounced as in the domains discussed above. Numerous researchers found no clear differences in the environmental domain scores between yogis and people who did not practise yoga [17,26,28]. Therefore, the discrepancies in the obtained results may be related to both the diversity of the SG and the CG and the selection of yoga techniques used in the interventions.

Even though the study group obtained higher scores than the control group, we observed that the social domain of the quality of life was the only one in the presented study whose scores did not differ among both groups. However, the obtained results contradict the results presented by other authors. Differences between groups of individuals with diagnosed diabetes [17] and residents of nursing homes [16] studied using the WHOQOL-BREF questionnaire were substantial. Furthermore, studies using other questionnaires revealed significant differences in the scores in this domain [18,20,21]. It is worth noting that participants of all the mentioned studies were much older than the participants of our study, which consequently could have had an impact on their quality-of-life scores in the social domain. It can also be assumed that the feeling of unity during yoga classes strengthens social ties among people who face similar difficulties and limitations resulting from age and illness. The inconsistency of the results of our research with the reports of other authors may also be related to the variety and selection of yoga techniques used in interventions.

In our research, we also found statistically significant differences in terms of quality-of-life satisfaction and own health satisfaction. Several studies that used the WHOQOL-BREFF questionnaire yielded similar results, thus indicating the high quality of life satisfaction among yogis. Oftentimes these results were accompanied by other objective changes, such as lower blood pressure [28]; differences in terms of evaluation of positive and negative symptoms of an illness [26]; decreased menopausal symptoms, stress level and depressive symptoms [29]; and even a tendency to improve glycaemic control [17]. 

The results of our research are positively associated with the studied variables. The selection of study participants largely limited the influence of factors other than yoga exercises that could be related to the quality-of-life level. However, inclusion criteria for the study group allowed for a wide variety of implementation of this form of activity by its members. For this reason, the authors decided to use the information collected through the interview questionnaire to characterise the type of undertaken exercises and determine the relationship between the characteristics of yoga practice and quality-of-life determinants. Moreover, the authors assumed that the quality-of-life level is associated with yoga practice experience and the frequency of the undertaken activity. 

Our analysis showed statistically significant differences in quality-of-life levels in somatic, psychological, and social domains between groups with varying yoga practice experiences. Nevertheless, adults who have been practising yoga for less than two years scored lower than adults who have been practising yoga for more than ten years only in the somatic domain. No similar relationships were found between other groups with varying yoga practice experiences. A similar relationship was observed by Bridee et al., whose research revealed a relation between yoga practice experience and the physical health domain assessed using the PROMIS Global 10. These results allowed us to conclude that there is a significant relationship between long-term yoga practice and better physical health, which has the potential to impart added health benefits over time [30]. This view also seems to be valid in the context of a study conducted on the population of Australian yogis, whose results revealed a clear relationship between yoga exercises and the quality-of-life score in five domains. By analysing separately groups of practitioners and yoga teachers, we found even two-fold differences in assessments of the positive impact of the said activity on quality-of-life domains. Furthermore, the authors also determined that yoga teachers practised yoga significantly more frequently, and they had more practice experience [23]. Results of Hewett’s research on adults under stress leading a sedentary lifestyle confirm there is a relationship between commitment to yoga practice and health outcomes. Even though practitioners of *Bikram Yoga* scored higher than the control group in health-related quality-of-life domains, we observed that practice attendance correlated positively with scores in the quality-of-life domains [31]. 

Given the multiplicity of yoga practice characteristics, including practice duration and frequency, paths, performed practices, and the degree of commitment, it can be assumed that it is difficult to clearly estimate the extent and impact of yoga exercises on an individual. Therefore, it appears reasonable to conclude that all the above factors may determine the effectiveness of yoga exercises, and thus differentiate health benefits of yoga practice. Although there are no proven relationships between the quality-of-life level and the frequency of yoga practice, the above information suggests that commitment to yoga practice can significantly affect achieved health benefits and quality of life. However, results of the current research and literature analysis suggest that the extent of achieved health benefits is tied to long-term behavioural changes, rather than short-term and intensive activities. Thus, yoga’s cumulative health benefit over time, also mentioned in our study [30], as well as the tendency for spill-over behaviour effects to occur [32], and centuries-old yoga practices confirm Patanjali’s thesis that one’s confidence about yoga may impact the likelihood of improved health gains from yoga practice [33]. 

## 5. Conclusions

Yoga practice is associated with a higher quality-of-life score in somatic, psychological, and environmental domains and a greater quality of life satisfaction and own health satisfaction in healthy adults. Furthermore, long-term yoga practice is positively associated with the level of quality of life in the somatic domain.

## Figures and Tables

**Table 1:** Comparative characteristics of socio-demographic data of all study participants.

Variable		SG		CG		*p*
		*N*	*%*	*N*	*%*	
**Sex**	Female	124	82.7	124	82.7	1.000 ^a^
	Male	26	17.3	26	17.3	
	Total	150	100.0	150	100.0	
**Age**	*M*	42.53		41.43		0.053 ^b^
	*SD*	4.697		4.729		
	*Me*	43.0		40.0		
	*Min*	35.0		31.0		
	*Max*	50.0		51.0		
	*Q1*	38.0		37.0		
	*Q3*	46.0		45.0		
**Family status**	Married	105	70.0	122	81.3	0.057 ^a^
	Cohabitation	17	11.3	13	8.7	
	Single	28	18.7	15	10.0	
	Total	150	100.0	150	100.0	
**Place of residence**	Village	18	12.0	32	21.3	0.057 ^a^
	City with a population under 10,000	4	2.7	10	6.7	
	City with a population between 10,000 to 50,000	13	8.7	11	7.3	
	City with population between 50,000 to 100,000	10	6.7	5	3.3	
	City with a population above 100,000	105	70.0	92	61.3	
	Total	150	100.0	150	100.0	

Annotation. SG—study group; CG—control group; *M*—medium, *SD*—standard deviation; *Me*—median; *Min*—minimum value; *Max*—maximum value; *Q1*—lower quartile; *Q3*—upper quartile; *p*—statistical significance; ^a^—chi-squared test; ^b^—Mann–Whitney U-test.

**Table 2:** Comparative characteristics of education of both the SG and the CG.

			Group		Test Result
			Study Group	Control Group	
**Education**	Higher	*N*	127	101	χ^2^ = 11.422 *df* = 1 *p* = 0.001
		%	84.7	67.3	
	Other than higher	*N*	23	49	
		%	15.3	32.7	
Total		*N*	150	150	
		%	100.0	100.0	

Annotation. *p*—significance; *df*—degrees of freedom; χ^2^—test.

**Table 3:** Comparative characteristics of determinants of quality of life, quality of life satisfaction, and own health satisfaction in both the SG and CG.

	WHOQOL 1		WHOQOL 2		WHOQOL 3		WHOQOL 4		WHOQOL 5		WHOQOL 6	
	SG	CG	SG	CG	SG	CG	SG	CG	SG	CG	SG	CG
*M*	16.99	15.74	15.01	14.03	15.29	15.31	15.20	14.21	4.15	3.97	4.03	3.70
*SD*	1.85	2.32	2.17	2.41	2.85	2.73	1.81	2.04	0.62	0.68	0.64	0.89
*Min*	10.86	8.57	9.33	8.00	5.33	8.00	9.50	8.00	2.00	1.00	2.00	1.00
*Max*	20.00	20.00	20.00	20.00	20.00	20.00	19.00	19.50	5.00	5.00	5.00	5.00
*Q25*	16.00	14.29	14.00	12.00	13.33	13.33	14.00	13.00	4.00	4.00	4.00	3.00
*Me*	17.14	15.71	15.33	14.00	16.00	16.00	15.50	14.00	4.00	4.00	4.00	4.00
*Q75*	18.29	17.14	16.67	15.33	17.33	17.33	16.50	15.50	5.00	4.00	4.00	4.00
*U*	7579.00		8554.00		11141.50		7919.50		9794.50		9194.50	
*p*	<0.001 *		<0.001 *		0.884		<0.001 *		0.023 *		0.002 *	

Annotation. SG—study group; CG—control group; WHOQOL 1—quality of life in the somatic domain; WHOQOL 2—quality of life in the psychological domain; WHOQOL 3—quality of life in the social domain; WHOQOL 4—quality of life in the environmental domain; WHOQOL 5—quality of life satisfaction; WHOQOL *6*—own health satisfaction; *M*—medium; *SD*—standard deviation; *Min*—minimum value; *Max*—maximum value; *Q25*—lower quartile; *Me*—median; *Q75*—upper quartile; *U*—Mann–Whitney U-test statistics; *p*—Mann–Whitney U-test significance; *—*p* < 0.05.

**Table 4:** Comparative characteristics of quality-of-life levels in several domains of groups with varying yoga practice experience.

Scale	Test Result
**WHOQOL 1**	*χ*^2^ = 9.957 *df* = 4 *p* = 0.041 *
**WHOQOL 2**	*χ*^2^ = 9.636 *df* = 4 *p* = 0.047 *
**WHOQOL 3**	*χ^2^* = 11.129 *df* = 4 *p* = 0.025 *
**WHOQOL 4**	*χ*^2^ = 4.153 *df* = 4 *p* = 0.386

Annotation. WHOQOL 1—quality of life in the somatic domain; WHOQOL 2—quality of life in the psychological domain; WHOQOL *3*—quality of life in the social domain; WHOQOL 4—quality of life in the environmental domain; *χ*^2^—ANOVA Kruskal–Wallis test statistics; *p*—test significance; *—*p* < 0.05.

**Table 5:** Comparative characteristics of quality of life in groups with varying frequency of yoga practice.

Scale	Test Result
**WHOQOL 1**	*χ*^2^ = 5.886 *df* = 2 *p* = 0.053
**WHOQOL 2**	*χ*^2^ = 4.484 *df* = 2 *p* = 0.106
**WHOQOL 3**	*χ*^2^ = 1.053 *df* = 2 *p* = 0.591
**WHOQOL 4**	*χ*^2^ = 1.407 *df* = 2 *p* = 0.495

Annotation. WHOQOL 1—quality of life in the somatic domain; WHOQOL 2—quality of life in the psychological domain; WHOQOL 3—quality of life in the social domain; WHOQOL 4—quality of life in the environmental domain; *χ*^2^—Kruskal–Wallis test statistics; *p*—test significance.

## Data Availability

Data supporting the findings of this study are available from the corresponding author upon request.
